# Nominal plan robustness may predict plan degradation in proton therapy for oropharyngeal head‐and‐neck cancer

**DOI:** 10.1002/acm2.70585

**Published:** 2026-05-01

**Authors:** Mark J. Zakhary, Daniel Redell, David Alicia, Jenna Jatczak, Nrusingh C. Biswal, Sina Mossahebi, Kai Sun, Reid Bark, Jason K. Molitoris, Byongyong Yi

**Affiliations:** ^1^ Department of Radiation Oncology University of Maryland School of Medicine Baltimore Maryland USA; ^2^ Maryland Proton Treatment Center Baltimore Maryland USA; ^3^ University of Maryland College Park Maryland USA; ^4^ Division of Biostatistics and Bioinformatics University of Maryland Baltimore Baltimore Maryland USA

**Keywords:** adaptive radiation therapy, head‐and‐neck cancer, pencil beam scanning, proton therapy, proton treatment planning, verification CT

## Abstract

**Background:**

Proton therapy for head‐and‐neck (HN) cancer offers superior organ‐at‐risk sparing compared to photon therapy, but is challenged by frequent anatomical changes during treatment. These changes need to be monitored with routine verification CTs (vCTs), which are used to trigger adaptive replans when deemed necessary by the clinical team.

**Purpose:**

To investigate whether nominal plan robustness evaluation (RE) data—specifically the magnitude and spatial characteristics of high‐dose regions (hotspots)—can predict the development of clinically significant hotspots on verification CTs (vCTs) and guide planning strategies that minimize the need for adaptive replanning.

**Methods:**

This retrospective study analyzed 46 patients with p16‐positive oropharyngeal cancer treated with proton therapy. Clinical treatment plans were robustly evaluated using 12 uncertainty scenarios combining 3 mm setup and ± 3.5% range errors. Each plan was recalculated on periodic vCTs throughout the treatment course to assess plan degradation. The maximum RE hotspot magnitude and location were compared with vCT hotspot characteristics. A subset of five cases underwent proof‐of‐concept replanning to reduce RE hotspots and assess downstream vCT dose effects.

**Results:**

Patients requiring adaptive replanning due to vCT hotspots had significantly higher RE hotspot magnitudes of the nominal plan compared to those who did not (*p* = 0.008). For replanned cases, higher RE hotspots were moderately correlated with closer proximity of RE and vCT hotspots (*r* = −0.59, *p* = 0.009). Across all patients, a modest correlation (*r* = 0.58, *p* < 0.001) was observed between RE and vCT hotspot magnitudes. Further, the rate of plan degradation over the course of treatment via hotspot formation was found to increase with increasing RE hotspot magnitude. Replanning to reduce RE hotspots led to an average 5.6% reduction in vCT hotspot dose for the five patients studied, suggesting that reducing RE hotspots may reduce the frequency of replans.

**Conclusions:**

Nominal plan robustness evaluation is predictive of both the magnitude and location of hotspots observed on vCTs, and plans with higher RE hotspots tend to degrade faster over the treatment course. Minimizing RE hotspots during treatment planning may reduce the need for adaptive replanning and enhance clinical workflow efficiency.

## 1 INTRODUCTION

The use of proton therapy for the treatment of head‐and‐neck (HN) cancer is becoming increasingly commonplace.^1^, ^2^ Proton therapy can reduce integral dose and dose to critical OARs compared to photon therapy.^3^, ^4^ Alongside these significant advantages come substantial challenges. In particular, HN cancer is prone to changes during the course of treatment, such as weight loss, tumor growth or shrinkage, and airway changes.^5^, ^6^, ^7^ Consequently, routine verification CTs (vCTs) must be acquired throughout the treatment course in order to evaluate how well the clinical plan is holding up as the patient's anatomy changes.^8^, ^9^, ^10^ If changes are substantial and the clinical goals are no longer met, a revision plan must be generated. HN is among the disease sites requiring revision most frequently during the treatment course, a time‐intensive process.^11^, ^12^

**FIGURE 1:**
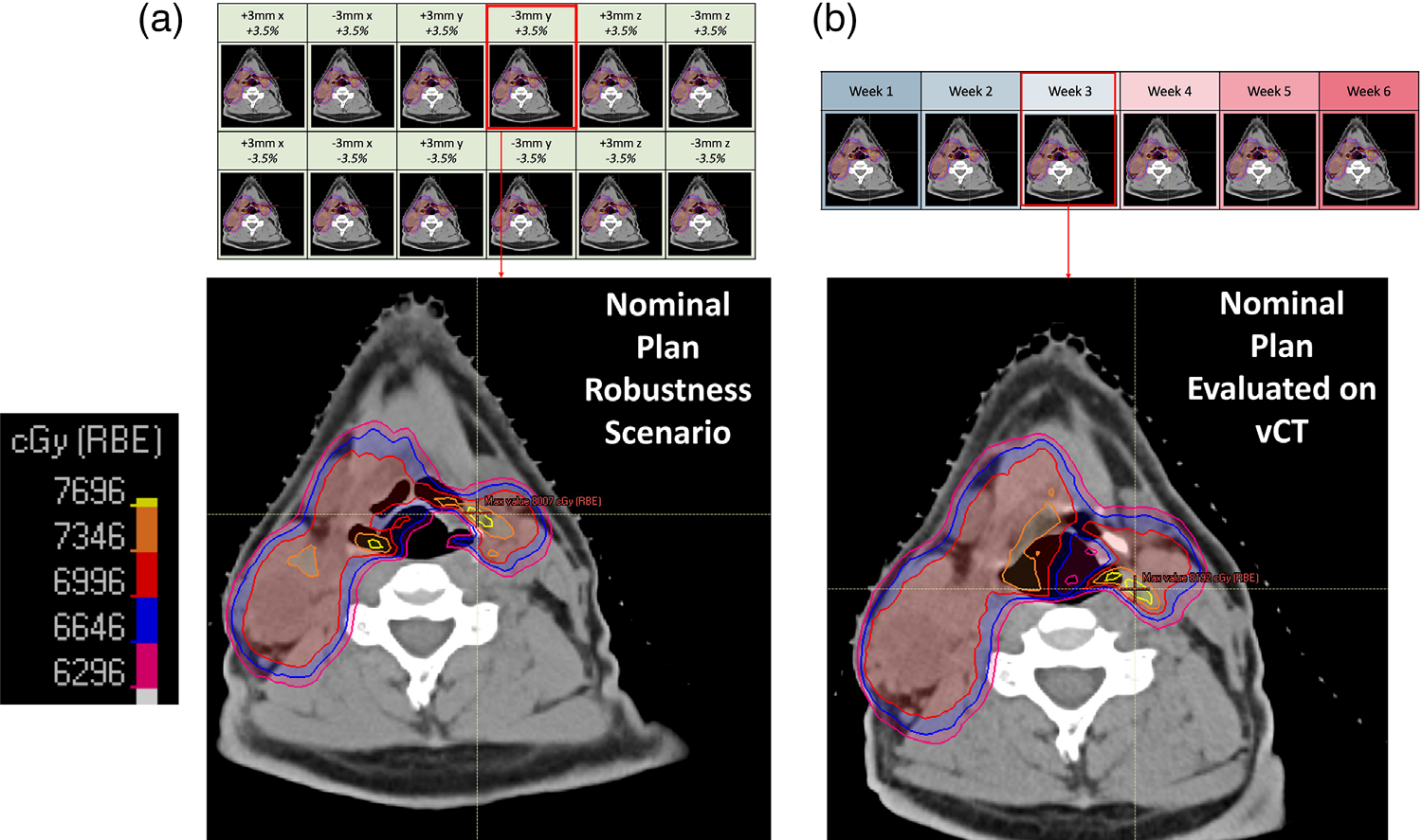
An example of a RE hotspot predicting a vCT hotspot. In panel (a), a hotspot is identified in one of the robustness evaluation scenarios near the airway with a magnitude of about 80 Gy, or about 114% of the highest prescription dose level for this patient. In panel (b), a hotspot of similar magnitude (82 Gy) in a similar location is identified when the clinical plan is reevaluated on a vCT scan. In this particular case, the attending physician elected to replan the case because of the vCT hotspot.

As a result of these challenges, significant efforts have been put forth to improve the efficiency and efficacy of HN proton therapy, such as on‐line adaptation, CBCT‐based dosimetric evaluation, and various robust planning techniques, among other strategies.^5^, ^7^, ^13^, ^14^, ^15^ Accounting for these unpredictable patient‐specific anatomical changes a priori during treatment planning is challenging. In this report, we demonstrate that information gleaned in the robust evaluation of a HN proton plan prior to treatment can be predictive of subsequent revision planning. Specifically, the magnitude and location of high‐dose voxels (“hotspots”) in the various robustness evaluation (RE) scenarios can be predictive of hotspots found on vCT‐evaluated plans (“vCT plans”) that ultimately necessitate a revision plan (Figure 1). This finding suggests that plan degradation typically attributed to anatomical change may actually be due in large part to suboptimal plan robustness, and thus potentially preventable. The correlation between RE and vCT hotspots can help elucidate potential promising treatment planning strategies that would reduce the likelihood of hotspots developing throughout the treatment course, thereby potentially improving dosimetric integrity and clinical workflow efficiency. Because hotspots are among the most frequent causes of revision planning for HN proton therapy at our institution, identifying strategies that could reduce their incidence could have a significant clinical impact.

## 2 METHODS

Under an IRB approved protocol, 46 patients with p16+ oropharyngeal cancer who were treated with definitive intent using proton radiotherapy to the bilateral neck between May 2019 and November 2022 were analyzed for this study. Demographic information, treatment plan parameters, and replan information were collected for all patients. All patients were prescribed to 70 Gy, and all but two patients received the prescription dose. Two patients in the replanned group received 63.5 Gy and 67.8 Gy, respectively; these two patients were unable to complete their course of treatment due to nausea. All patients had an ECOG performance status of 0–1. CTVs were defined using gross disease, both primary and gross lymph nodes, plus a 0–5 mm margin for the full dose volumes. Lower dose CTVs included at risk or involved lymph node regions, predominantly levels II, III, IV and retropharyngeal regions. These lower dose regions ranged from 50 to 60 Gy depending on a variety of risk factors and whether or not patients were postoperative in those regions, therefore necessitating a higher dose to overcome lower oxygenation. In some cases, these lower dose levels were discrepant between ipsilateral and bilateral, depending on the features of the case. The volumes were treated via simultaneous integrated boosts. Each clinical plan utilized 4 or 5 beams using a multi‐field optimized (MFO) planning approach and was robustly optimized for CTV coverage using 3 mm setup uncertainty and 3.5% range uncertainty. Robustness was evaluated in 12 uncertainty scenarios, combining ± 3 mm setup uncertainty in a single cardinal direction with a ± 3.5% range error. Robust optimization objectives were selected to meet our institutional standard that a minimum of 95% of the CTV targets receive 95% of the prescription dose in the worst‐case robustness scenario, although in practice coverage often exceeds this threshold. Further robust optimization objectives to control hotspots were also included, typically aiming to keep the maximum dose in the worst‐case robustness scenario to below 110% of the highest prescription dose level. The RayStation (versions 8A, 11A, and 11B; RaySearch laboratories, Sweden) treatment planning system was used for plan generation and robustness evaluation (RE).

For each patient requiring a revision plan due to a hotspot on a verification CT, the magnitude and location of the hotspot were identified (to be referred to as “revision hotspots” throughout this report). Subsequently, for the original plan on the initial simulation CT, any hotspots within 5% of the magnitude of the revision hotspot were identified within the 12 robustness evaluation (RE) scenarios (to be referred to as “robust evaluation (RE) hotspots” throughout this report), as well as their locations relative to the revision hotspot. Finally, correlations were extracted based on the relative magnitude, location, and frequency of RE hotspots relative to the revision hotspot.

For all patients in this study, each treatment plan was recalculated on each of their vCTs, and the magnitude of the vCT hotspots was tabulated and compared against the maximum RE hotspots for those patients. vCT hotspots were evaluated even if the original plan had been retired, and the patient had moved on to a revision plan at the time of a given vCT. These data were also compared against the time elapsed from simulation to the date of each vCT.

**FIGURE 2:**
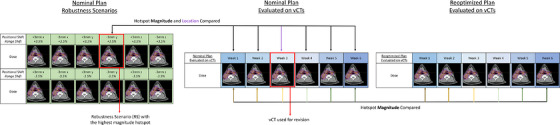
Analysis workflow illustration. For all patients treated at our institution, the nominal plan was evaluated in twelve robustness scenarios as part of routine clinical plan evaluation, as well as on each periodic vCT to verify sustained dosimetric integrity. For patients in this study, the magnitude of the highest hotspot among the 12 RE was compared to hotspots on each vCT plan. The location of the hotspots was also compared for the vCTs ultimately used for a revision plan. For a subset of patients, plans were reoptimized to try to reduce the maximum RE hotspot. These reoptimized plans were recalculated on each vCT, and the hotspots for each vCT plan was compared to the original vCT plan to investigate the effect of reoptimization on vCT plan hotspot magnitude.

For a small subset of five treatment plans with relatively high maximum RE hotspots (> 115% of the highest prescription dose level), a proof‐of‐concept replanning study was performed to evaluate the potential effect of reducing the magnitude of RE hotspots during planning on vCT hotspots. For each patient, the RE hotspots were reduced while maintaining similar dosimetry to the clinical plan. The modified plans were evaluated on each vCT, and the results were compared to those of the clinical plan. An illustration of the analysis workflow in this study is shown in Figure 2.

The student *t*‐test was used to calculate p values in this study, with p values below 0.05 being considered statistically significant. Correlation coefficients (*r*) were also calculated to quantify the strength of the correlations presented.

## 3 RESULTS

**FIGURE 3:**
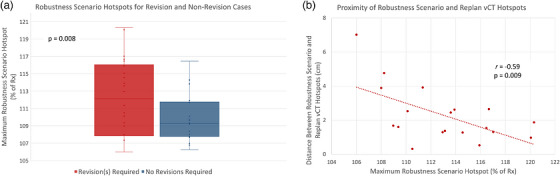
Hotspot magnitude and location comparison for revised and non‐revised plans. In panel (a), the magnitude of the maximum robustness scenario (RS) hotspot is compared between cases requiring at least one revision plan throughout the treatment course, and cases not requiring any revision plans. The maximum RE hotspot is significantly higher for the revision cases. In panel (b), the location of the maximum RE hotspot was compared to the location of the hotspot on the vCT used for revision planning, for the subset of cases revised specifically due to a hotspot. A negative correlation is observed, indicating that higher magnitude RE hotspots tend to lead to a closer spatial correlation between RE hotspots and vCT plan hotspots.

Patient demographic information is shown in Table 1. Of the patients included in this study, 19 required at least one revision plan due to an unacceptable hotspot throughout their treatment course, 8 required at least one revision plan for a different reason (undercoverage or setup changes), and 19 did not require a revision plan. The maximum RE hotspot for cases that had to be revised as well as for cases not requiring a revision plan are shown in Figure 3a. The maximum RE hotspots were significantly higher for revised cases compared to cases that did not require a revision (*p* = 0.008), suggesting a possible correlation between high RE hotspots and plan degradation. Further, for cases revised due to a hotspot, the data shows that as the maximum RE hotspot increases, the distance between the location of the maximum RE hotspot and the location of the revision vCT hotspot tends to shrink (*r* = −0.59, *p* = 0.009) (Figure 3b). This suggests that higher RE hotspots may be more spatially predictive of plan degradation.

**TABLE 1:** Patient demographics information.

	Replanned (*n* = 27)	Not replanned (*n* = 19)
Median age (range)	65 (47–77)	65 (44–83)
White	25 (93%)	18 (95%)
Black	2 (7%)	1 (5%)
Male	26 (96%)	18 (95%)
Female	1 (4%)	1 (5%)
Concurrent cisplatin (1 cycle every 3 weeks)	15 (56%)	7 (37%)
Weekly concurrent cisplatin	9 (33%)	5 (26%)
Other concurrent chemotherapy regimen	0 (0%)	3 (16%)
No chemotherapy	3 (11%)	2 (11%)
Base of tongue	14 (52%)	10 (53%)
Tonsil	11 (41%)	9 (47%)
Entire oropharynx	2 (7%)	0 (0%)
Mean GTV volume (standard deviation)	33.96 (27.46) cc	23.69 (32.41) cc
T1	0 (0%)	3 (16%)
T2	12 (44%)	10 (53%)
T3	5 (19%)	3 (16%)
T4	10 (37%)	3 (16%)
N0	1 (4%)	3 (16%)
N1	17 (63%)	8 (42%)
N2	7 (26%)	8 (42%)
N3	2 (7%)	0 (0%)
*X* beam arrangement	7 (26%	3 (16%)
Raven beam arrangement	17 (63%)	15 (79%)
5‐field	3 (11%)	1 (5%)

*Note*: “*X*” beam arrangement consists of two posterior oblique beams and two anterior oblique beams. “Raven” beam arrangement consists of an anterior beam, a posterior beam, and two anterior oblique beams. The 5‐field beam arrangement consists of either the “*x*” or the “Raven” beam arrangement, with the addition of an anterior beam kicked superiorly.

**FIGURE 4:**
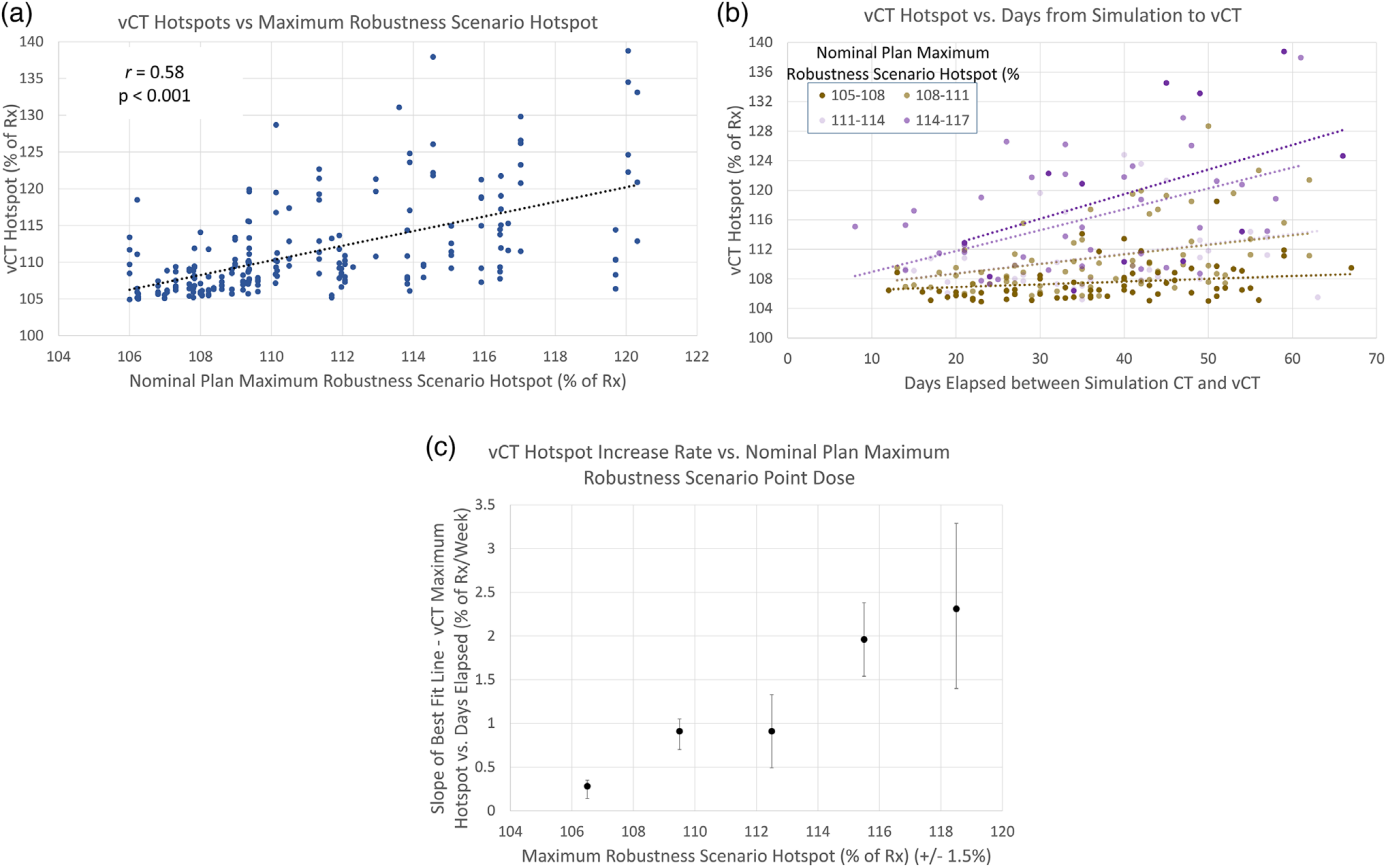
Correlation between robustness scenario hotspots and vCT Hotspots. In panel (a), a modest positive correlation is observed between vCT plan hotspots (*y*‐axis) and the maximum robustness scenario (RS) hotspot on the *x*‐axis. In panel (b), the magnitude of vCT hotspots are plotted against the days elapsed between simulation and vCT, with the magnitude of the maximum RE hotspot indicated via the color scale. This plot shows that the average rate of plan degradation, indicated by the slope of the dotted best fit lines, increases with increasing maximum RE hotspot. In panel (c), the slopes of the best fit lines are plotted against the maximum RE hotspot.

The analysis above relies on the attending physician's subjective decision to create a revision plan. To more objectively quantify the correlation between RE and vCT hotspots, a second analysis was performed for all patients in this study, regardless of whether they required a revision and the reason for the revision. All vCTs the patients received after the initiation of their treatment plan were included in this analysis, by recalculating each of their treatment plans on each vCT. For patients who received a plan revision, each plan revision was calculated on each subsequent vCT. Figure 4a shows the magnitude of vCT hotspots plotted against the maximum RE hotspots, showing a modest correlation (*r* = 0.58, *p* < 0.001). In Figure 4b, vCT hotspots are plotted against the days elapsed between the simulation CT and vCT, with the maximum RE hotspot delineated via the color scale. The best‐fit lines demonstrate that as the maximum RE hotspot increases, the vCT hotspots tend to increase at a faster rate throughout the treatment course. This again points to a correlation between higher RE hotspots and faster plan degradation. The slopes of the best‐fit lines are extracted and plotted in Figure 4c, demonstrating that as the maximum RE hotspot increases, the plans tend to degrade faster. For cases with RE hotspots > 117% of the highest prescription dose, the rate of hotspot increase was highest (around 2.5% of Rx dose/day), indicating rapid plan degradation and a high likelihood of necessitating one or more revision plans.

A proof‐of‐concept study was performed for a subset of patients to evaluate whether reoptimizing plans with a focus on RE hotspots could reduce vCT hotspots. For five patients whose clinical plans exhibited maximum RE hotspots > 115% of the highest prescription dose level, plans were reoptimized to reduce the maximum RE hotspot without modifying any other optimization objectives. The reoptimized plans were recalculated on each vCT to evaluate the impact on vCT hotspots (Table 2). For the five reoptimized plans, the maximum RE hotspot was reduced by an average of 6.4% (range 3.2–8.3%), and vCT hotspots were reduced by an average of 5.6% (range 1.7% increase to 21.8% reduction). The dosimetry of the reoptimized plan was also compared to the original plan to evaluate the impact of the reoptimization on key target and OAR dosimetric parameters (Table 3). The parameters evaluated were not significantly changed due to the reoptimization, and the reoptimized plans would still meet our clinic's guidelines for acceptability.

**TABLE 2:** Results of a preliminary replanning study.

Plan number	Clinical plan maximum RE hotspot (% Rx)	Reoptimized plan maximum RE hotspot change (% Rx)	vCT 1 hotspot change (% Rx)	vCT 2 hotspot change (% Rx)	vCT 3 hotspot change (% Rx)	vCT 4 hotspot change (% Rx)	vCT 5 hotspot change (% Rx)	vCT 6 hotspot change (% Rx)
1	120.4	−6.8	−4.9	−11.3	−21.8	–	–	–
2	115.9	−3.2	1.7	0.6	−5.5	−4.9	−5.8	−6.1
3	120.1	−8.3	−0.5	−7.8	−10.8	−3.0	–	–
4	117.0	−7.2	−1.1	−5.5	−12.3	−5.4	−10.5	−5.7
5	116.5	−6.4	0.7	−2.1	−2.8	−2.7	−3.7	–

*Note*: For a subset of patients, plans were reoptimized by dosimetry staff to reduce the maximum robust evaluation (RE) hotspot. These reoptimized plans were then evaluated on each vCT, and the maximum hotspot of each vCT plan was compared to the original vCT evaluation using the clinical plan. The percentage changes indicated show the difference between the original plan hotspot and the reoptimized plan hotspot. The reoptimized plans successfully reduced the maximum RE hotspots by an average of 6.4% of the prescription dose, resulting in an average vCT plan hotspot reduction of 5.6% of the prescription dose.

**TABLE 3:** Dosimetric comparison between reoptimized and clinical plans.

Target/OAR metric	Clinical plan mean [range]	Reoptimized plan mean [range]	Absolute change mean [range]	Percent change mean [range]
CTV_high worst case D95% (%)	98.2 [97.5, 98.7]	98.1 [97.4, 98.5]	0.0 [−0.2, 0.2]	0.0 [−0.2, 0.2]
CTV_intermediate worst case D95% (%)	98.1 [97.0, 98.8]	98.0 [96.2, 98.9]	0.1 [−1.3, 0.8]	0.1 [−1.3, 0.9]
CTV_low worst case D95% (%)	98.0 [96.1, 99.6]	98.1 [96.5, 99.7]	−0.17 [0.5, 0.1]	−0.17 [−0.5, 0.1]
Mandible maximum point dose (Gy)	71.3 [70.5, 72.2]	71.4 [70.6, 72.1]	0.1 [−1.1, 1.5]	0.2 [−1.5, 2.1]
Brainstem maximum point dose (Gy)	29.2 [14.7, 43.4]	29.5 [14.5, 42.4]	0.3 [−3.3, 5.0]	4.7 [−8.9, 33.3]
Oral cavity mean (Gy)	16.8 [9.8, 28.0]	17.2 [10.0, 27.9]	0.4 [−0.4, 2.6]	3.4 [−2.1, 18.4]
Larynx mean (Gy)	29.5 [29.4, 29.6]	27.8 [24.3, 29.6]	−1.7 [−5.2, 0.2]	−5.6 [−17.6, 0.7]
Ipsilateral partoid mean (Gy)	32.0 [25.3, 39.7]	32.3 [25.4, 40.0]	0.3 [0.1, 0.9]	1.1 [0.3, 2.9]
Contralateral parotid mean (Gy)	22.8 [12.0, 33.7]	23.8 [12.0, 33.8]	1.0 [0.0, 4.3]	7.8 [0.0, 35.8]
Pharyngeal constrictor mean (Gy)	40.0 [33.9, 50.4]	37.6 [28.2, 52.2]	−2.3 [−9.4, 1.8]	−6.6 [−25.0, 3.6]
Spinal cord maximum point dose (Gy)	32.0 [23.2, 37.1]	31.8 [23.7, 35.4]	−0.1 [−2.1, 2.4]	−0.2 [−6.1, 7.3]

*Note*: For the cases reoptimized for the replanning study, the dosimetric parameters between the reoptimized plans and clinical plans were compared, to evaluate the impact of reducing RE hotspots on other plan quality metrics. For the CTV targets, the worst‐case D95% coverage amongst the 12 evaluated robustness scenarios was compared between the reoptimized and clinical plans (first 3 rows). Key OAR metrics which impacted plan optimization were also compared. Dosimetric parameters were well preserved through the reoptimization, and all plans remained clinically acceptable per departmental guidelines after reoptimization.

## 4 DISCUSSION

In this study, we demonstrated that in cases replanned due to a hotspot, the magnitude and location of hotspots identified in RE seem to foreshadow hotspots observed in the vCT plan evaluation. These vCT hotspots are generally attributed primarily to anatomical change, with the underlying assumption that such changes are unpredictable and thus difficult to account for during the treatment planning phase. Analyzing the entire patient cohort, we found that RE hotspots are modestly correlated with vCT hotspots and that the rate of plan degradation via hotspot formation is higher for cases with higher RE hotspots. This suggests that reducing RE hotspots during initial plan optimization could help delay or prevent adaptive replans due to hotspot formation on vCT. For the five cases thus reoptimized in our proof‐of‐concept analysis, vCT hotspots were found to be substantially reduced on average.

Theoretically, RE and vCT evaluations are probing different potential dose‐perturbing effects. RE evaluates setup and range uncertainty effects on the planning CT, while vCT plan evaluation quantifies the effect of anatomical changes such as weight loss, tumor growth/shrinkage, or air cavity changes on dosimetric integrity.^16^ However, it is not altogether surprising that these two evaluations were found to be correlated. For example, if RE demonstrates that the positive range uncertainty scenarios tend to produce hotspots for a particular plan, a similar effect could be observed in a vCT plan due to tumor shrinkage reducing the water‐equivalent proton path length traversed by one or more beams. Therefore, the presence of hotspots in RE could signify that a particular plan is generally not robust to hotspots arising from many potential sources. Although such a correlation can thus be understood theoretically, it would not be expected to be perfect, as ultimately different types of treatment changes will perturb the dose distribution differently. The modest correlation we observed between RE and vCT hotspot magnitude and location reflects this limited relationship between RE and vCT evaluation.

In our clinical experience, RE hotspots are more prevalent for MFO (multi‐field optimized) plans compared to SFO (single‐field optimized) plans. This is because when each field delivers a relatively uniform dose to the target (SFO plans), modest setup variations, range variations, intrafraction motion, or anatomical changes tend not to cause substantial dose perturbations. On the other hand, for MFO plans which are highly modulated, often exhibiting relatively high dose gradients within each field, even modest changes can cause quite dramatic dosimetric degradation to the overall plan dose, when the delicate dose balance between the fields is disturbed.^17^ While MFO plans offer the optimizer substantially improved latitude to balance target coverage and OAR sparing, the price can be paid in plan degradation and revision planning down the road.

In practice, MFO plans’ lack of robustness can prove challenging or impossible to improve solely via fine‐tuning optimization objectives. In many cases, based on the beam angles, beam blocks, and the specific patient anatomy, clinically acceptable plans that meet the target and OAR constraints can preclude reducing RE hotspots beyond a point. Further investigation is needed to determine what planning techniques such as beam selection, beam block design, SFO/MFO hybrid technique, dose gradient reduction, and so forth could help improve plan robustness and thereby potentially minimize the frequency of vCT plan hotspots necessitating revision planning.

Based on these findings, our clinic has successfully implemented more stringent constraints on the maximum RE hotspot during routine plan evaluations for HN cases treated with proton therapy. Further analysis is needed to determine if these changes have reduced the plan revision rate for this disease site. Proton practices could potentially benefit by examining if such correlations exist within their clinical practice and if so, implementing similar interventions to reduce the rate of plan adaptation.

Several limitations to this study bear mentioning. The decision to proceed with plan revision based on vCT plan evaluation (Figure 3) is subjective based on each physician's judgment. To limit physician variability, we performed the analysis in Figure 4. Additionally, as discussed previously, the extent of correlation between RE and vCT plan hotspots is limited because severe anatomical changes can produce drastic effects on the dose distribution, and these changes simply cannot be predicted or accounted for a priori. Finally, patient‐specific factors such as demographics, beam approach, and GTV volume could also affect the correlations investigated here, and although some qualitative variations can be observed, these factors were not quantitatively analyzed here.

Future work on this topic could include a more extensive replanning study and an analysis of which plan features and associated optimization objectives best improve plan robustness, as well as an evaluation of the consequences of improved robustness on plan quality (e.g. higher integral dose). Further, this analysis can be extended to other HN subsites as well as to other disease sites, particularly those requiring more frequent plan adaptation. Additionally, machine learning tools could be used to efficiently identify other signatures within the nominal plan, which can predict plan degradation.^18^

## 5 CONCLUSIONS

We demonstrated that the magnitude and location of RE hotspots are moderately correlated with hotspots on vCT plans, and that the rate of plan degradation tends to be higher for plans with relatively high RE hotspots. Further, we presented preliminary evidence that reducing RE hotspots during initial planning can help plans hold up better to treatment changes, potentially preventing or delaying the necessity of creating a revision plan, and thereby improving dose fidelity and clinical efficiency. Further study is needed to determine what additional evaluation criteria and associated optimization techniques best improve the robustness of treatment plans, as well as to establish any similar correlations for other HN subsites and other disease sites.

## AUTHOR CONTRIBUTIONS

**Mark J. Zakhary**: Project administration; conceptualization; methodology; data collection; formal analysis; visualization; writing—original draft; manuscript review; and editing. **Daniel Redell**: Data collection; replanning study; and manuscript review. **David Alicia**: Data collection; replanning study; and manuscript review. **Jenna Jatczak**: Data collection; replanning study; and manuscript review. **Nrusingh C. Biswal**: Data collection; and manuscript review. **Sina Mossahebi**: Data collection; and manuscript review. **Kai Sun**: Statistical analysis; and manuscript review. **Jason K. Molitoris**: Project conceptualization; patient identification; and manuscript review. **Byongyong Yi**: Project conceptualization; methodology; writing—original draft; and manuscript review.

## CONFLICT OF INTEREST STATEMENT

The authors declare no conflicts of interest.

## ETHICAL APPROVAL

This work was approved by the institutional review board under IRB HP‐00080153 (GCC 1853).

## Data Availability

The data that support the findings of this study are available from the corresponding author upon reasonable request.
